# Primary leiomyosarcoma originating from the azygos vein misdiagnosed as tracheal tumor: a case report and literature review

**DOI:** 10.3389/fonc.2025.1639131

**Published:** 2025-10-22

**Authors:** Long-fei Zhu, Yu-jun Wang, Chun-jian Zuo, Yuan-lin Deng, Bin Jiang

**Affiliations:** ^1^ Department of Thoracic Surgery, Daping Hospital, Army Medical University, Chongqing, China; ^2^ Department of Pathology, Daping Hospital, Army Medical University, Chongqing, China

**Keywords:** leiomyosarcoma, azygos vein, surgery, case report, misdiagnose

## Abstract

Leiomyosarcomas are uncommon mesenchymal neoplasms. Primary mediastinal leiomyosarcomas are even rarer with only a limited number of cases reported. Given the high potential for recurrence and metastasis, accurate and timely diagnosis become extremely crucial for treatment. Herein, we present a unique case of a 70-year-old asymptomatic male patient with a primary leiomyosarcoma originating from the azygos vein, which was misdiagnosed as a tracheal tumor preoperatively. This report highlights the critical importance of including leiomyosarcoma in the differential diagnosis of mediastinal masses, especially when preoperative pathological findings indicate a spindle cell neoplasm of smooth muscle origin. Early recognition can more effectively assess intraoperative risks, optimize the surgical plan, and are essential to prevent locally invasive growth and potential metastasis. By analyzing the specific case and reviewing relevant literature, this study seeks to enhance our understanding of the diagnosis and treatment of primary mediastinal leiomyosarcoma, derive insights from our clinical experience, and emphasize the importance of maintaining suspicion and prudence among healthcare professionals.

## Introduction

Primary mediastinal leiomyosarcomas are rare tumors, accounting for less than 1% of all mediastinal neoplasms, while primary mediastinal leiomyosarcoma originating from the azygos vein is an extremely rare subtype (1, 2). Limited to a few single-institution studies and case reports on primary mediastinal leiomyosarcoma, the prognosis remains uncertain but is generally discouraging. Up to now, the sole nationwide series study reports that merely about 14.8% of the entire cohort survive for 5 years, whereas the 5-year survival rate is approximately 40.1% among patients who underwent R0 resections and received radiation therapy (3). Therefore, prompt and accurate diagnosis is crucial for improving the prognosis of patients. However, the lack of distinct clinical characteristics and the low incidence rate often lead to limited understanding among clinicians. Incorrect diagnosis can potentially lead to delays or errors in treatment, inadequate preparation for surgery, misjudgment of surgical risk, and ultimately result in a poor prognosis. In this study, we report a unique case of primary mediastinal leiomyosarcoma originating from the azygos vein confirmed by surgical resection and pathology which was misdiagnosed as a tracheal tumor preoperatively.

## Case presentation

**Figure 1:**
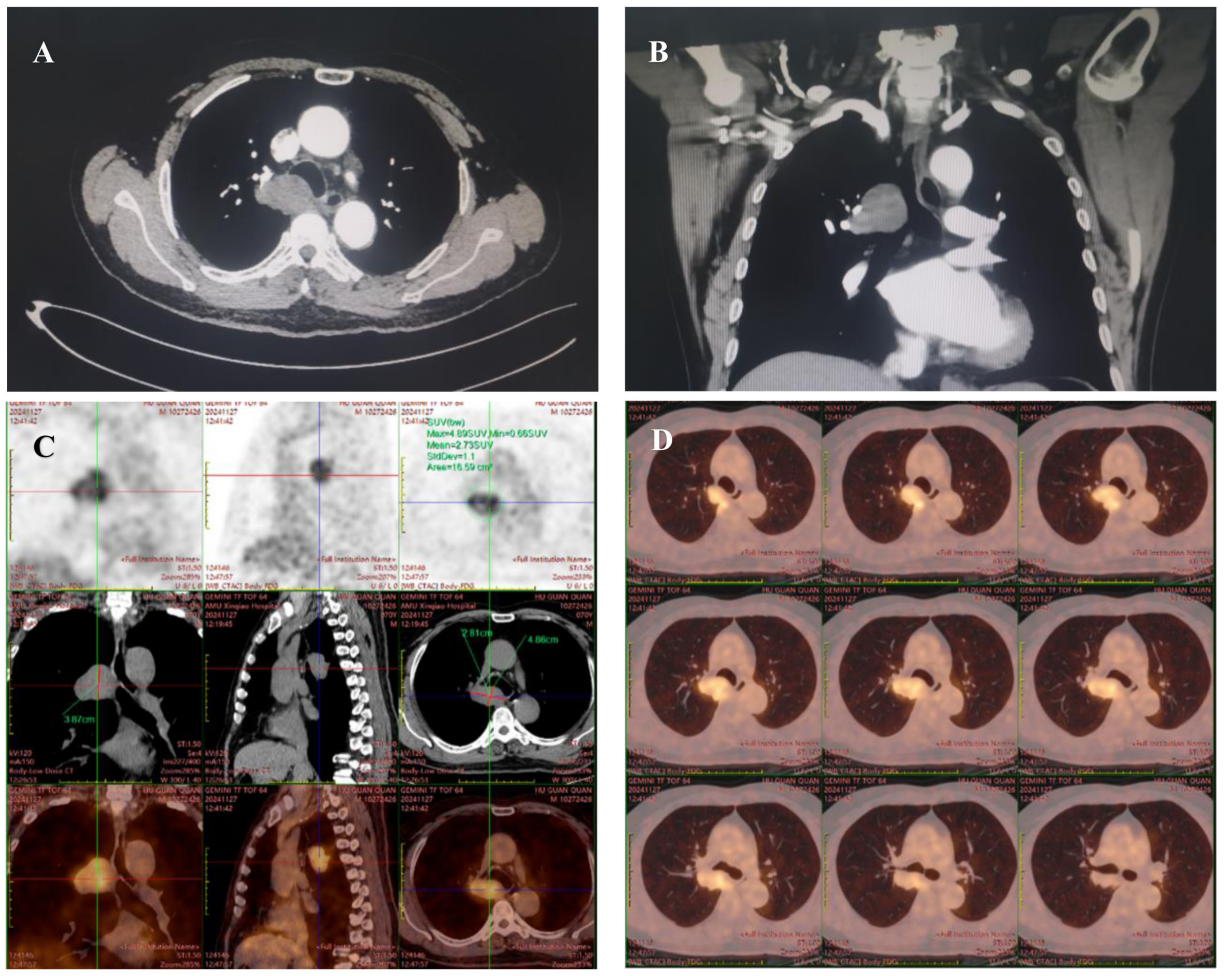
Chest computed tomography scan **(A, B)** reveals a 5.1 x 3.5 cm mass closely abutting the right wall of the lower tracheal segment and the right main bronchus. An 18F-FDG PET/CT scan **(C, D)**, extending from the vertex of the skull to the upper thighs for preoperative evaluation, revealed fluorodeoxyglucose accumulation in the tumor (maximum standardized uptake value, 4.9), suggesting a suspected malignancy.

A 70-year-old male patient with no known comorbidities or history of smoking or alcohol abuse was referred to our department for surgical evaluation of a mass in the upper-right part of the tracheal carina that was incidentally detected on thoracic computed tomography during a routine health check-up. Enhanced chest CT scanning revealed a circular and homogeneous mass measuring 5.1 x 3.5 cm in the middle mediastinum adjacent to the hilar region of the right lung, demonstrating heterogeneous and gradual enhancement. The mass was closely associated with the right wall of the lower tracheal segment and the right main bronchus felt to represent a tracheal tumor. The upper pole of the tumor was 3 cm above carina level and the azygos vein was compressed (**Figures 1A, B**). A fluorodeoxyglucose F18 positron emission tomography (PET) scan showed abnormal uptake in the mass (maximum standardized uptake value [SUVmax]: 4.9), suggesting a malignant tumor, while no distant metastasis was identified (**Figures 1C, D**). To assess the spatial relationship of the tumor relative to the esophageal wall, endoscopic ultrasound (EUS) was employed. And the result revealed hypoechoic lesions external to the wall of the mid-esophagus, characterized by irregular margins and heterogeneous internal echoes, yet the layers of the esophageal wall remained distinctly demarcated and intact. Bronchoscopy was performed, revealing an extramural compression covered by normal mucosa in the membranous portion of the lower trachea, located just above the carina. Endobronchial ultrasound-guided transbronchial needle aspiration (EBUS-TBNA) results showed a spindle cell neoplasm of smooth muscle cell origin; however, a definitive diagnosis could not be established based on these findings alone.

For diagnosis and treatment, thoracoscopic resection was performed through a three-port method. During the surgery, it was observed that the round-shaped tumor with relatively regular margins. Since it resulted in significant compression and narrowing of the azygos vein and was inseparable from the vein, the neoplasm was hypothesized to originate from the arch of the azygos vein. The most critical tight adhesions of the mass were found with the parietal pleura adjacent to the spine at the level of the arch of the azygos vein; however, it did not attach or directly invade the esophagus, trachea and right main bronchus, or the right lung. The neoplasia was then progressively separated from the posterior chest wall by gentle dissection and thickened parietal pleura near the tumor was completely resected as well. Fortunately, we noticed that the mediastinal mass did not extended into the spinal canal. The azygos vein was transected with a stapling device during thoracoscopy because of the tight attachment and extensive infiltration at the arch of the azygos vein.

The patient had an uncomplicated course after surgery and was discharged on postoperative day 5. Postoperative radiotherapy was launched 4 weeks after surgery. The prescription dose: CTV (tumor bed): 60 Gy/2 Gy/30f. After 7 months of follow-up, there were no signs of recurrence.

**Figure 2:**
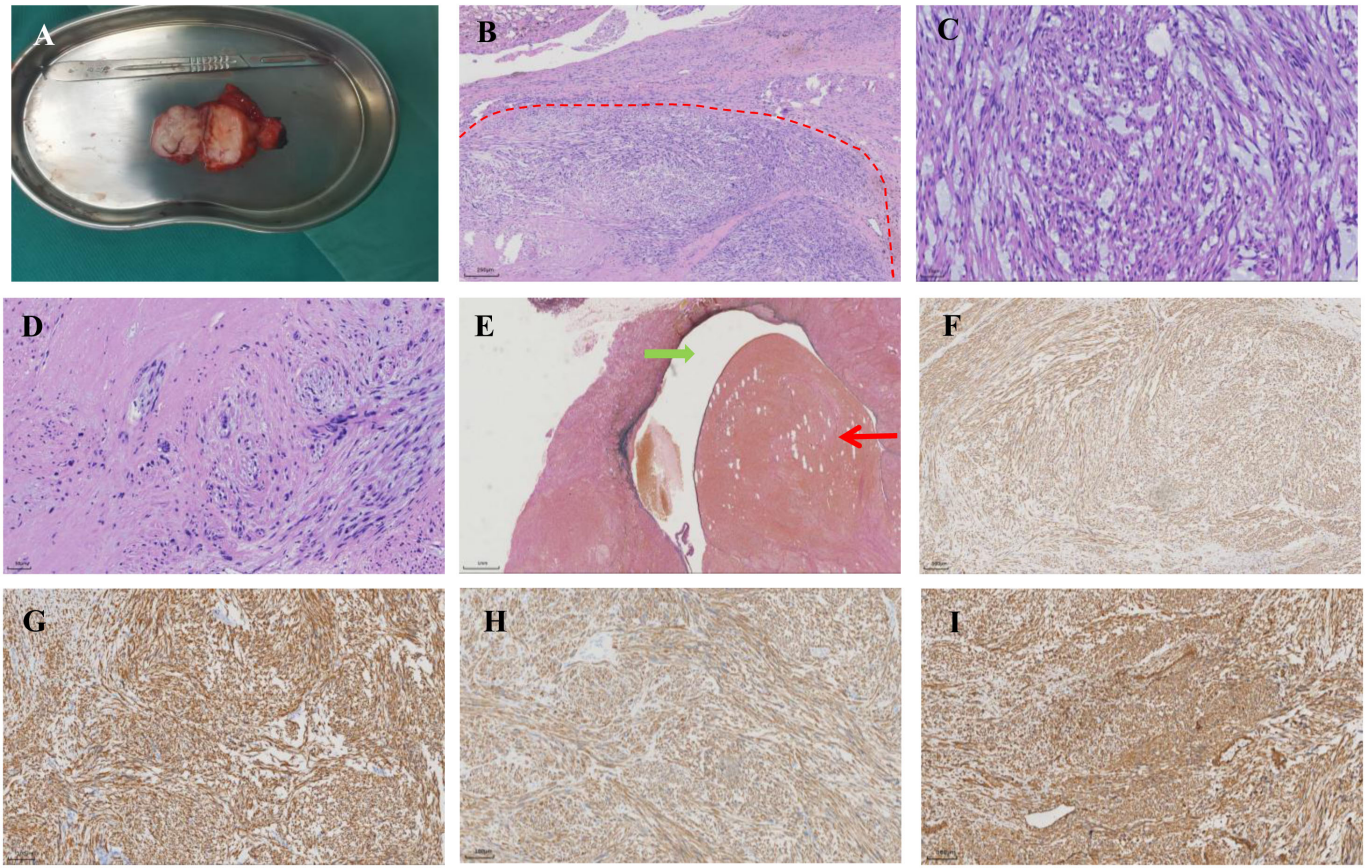
Pathological examination of the tumor. **(A)** Macroscopic image of the resected tumor. **(B)** Hematoxylin-eosin staining shows the spindle cell neoplasm (x 5). The red dashed line delineates the transitional zone between the tumor and adjacent normal tissue. **(C)** The area of mucoid degeneration in the interstitial tissue (x 20). **(D)** The presence of bizarre pleomorphic cells was observed (x 20). **(E)** Leiomyosarcoma arises from the wall of the azygos vein. The green arrow denotes the lumen of the azygos vein. Red arrow indicates the cancerous emboli within the vein (x 5). **(F–I)** The tumor is immunoreactive for smooth muscle actin, desmin, caldesmon, and vimentin respectively (x 10).

Macroscopically, the resected tumor was 6.8 cm in diameter, yellowish white, and solid (**Figure 2A**). Histopathologic examination revealed moderate to high atypia of the spindle cells, multiple foci of degeneration and necrosis (coagulative necrosis < 50%), and partial mucoid degeneration in the interstitial tissue (**Figures 2B, C**). Numerous bizarre pleomorphic cells and about 6-8 mitoses per high-power field were also present (**Figure 2D**). The tumor involved the azygos vein and exhibited invasion into adjacent adipose tissue and nerve fiber bundles, with evidence of cancerous emboli within the vein (**Figure 2E**). And the resection margins were negative. Immunohistochemical study showed strong diffuse staining for smooth muscle actin (SMA), desmin, caldesmon and vimentin (**Figures 2F–I**). The vascular endothelial marker CD34 was expressed and considered to be a positive finding, but it was focal. Results for cytokeratin, S-100 protein, CD21, and CD117 was negative. Staining for Ki-67 was focal, and its labeling index was about 60%. Based on these histopathological findings, leiomyosarcoma (Federation Nationale des Centers de Lutte le Cancer Grade 3, pT2b) was confirmed.

## Discussion

This study describes a mediastinal leiomyosarcoma originating from the azygos vein, accompanied by intra-venous tumor thrombus, which was misdiagnosed as a tracheal tumor preoperatively. For all we know, only three cases of leiomyosarcoma originating from the azygos vein have been previously documented (4–6).

The clinical presentation of primary mediastinal leiomyosarcomas is influenced by factors including tumor mass, origin, localization, size, and infiltration into adjacent tissues. Typically, patients are asymptomatic, and these tumors are often identified incidentally during routine health screenings or evaluations for unrelated conditions. However, in other cases, patients may exhibit non-specific symptoms including coughing, chest pain, back pain, and dyspnea. In the computerized tomography, primary mediastinal leiomyosarcoma typically presents as a lesion of equal or slightly lower density, with an ill-defined margin and a lobulated soft tissue density shadow. Following contrast enhancement, the tumor demonstrates heterogeneous enhancement, frequently accompanied by areas of necrosis and cystic changes (7). A PET/CT scan might help in the diagnosis by showing a high degree of 18F-2-fluoro-2-deoxy-D-glucose (FDG) uptake, however, its utility has also been questioned in some studies due to the limited overall specificity (8).

Unfortunately, the preoperative diagnosis of mediastinal leiomyosarcoma remains a great challenge due to nonspecific clinical and radiological features and the wide range of differential diagnoses that need to be considered. As in our instance, an overreliance on imaging findings led to our patient being misdiagnosed initially. The results of bronchoscopy and EBUS-TBNA biopsy were inconsistent with our expectations and fell outside the scope of presupposed differential diagnoses, leading to a period of confusion before the surgery. Although the primary treatments for both tracheal tumors and mediastinal leiomyosarcomas are radical resection, a relative accurate preoperative diagnosis can more effectively assess intraoperative risks, optimize the surgical plan, minimize complications, and thereby reduce perioperative risks, ultimately leading to improved patient outcomes. The misdiagnosis that occurs during the treatment process can primarily be attributed to the cognitive biases, such as the anchoring bias, where clinicians may overly rely on initial impressions or familiar patterns without adequately considering alternative diagnoses. Furthermore, although an intradepartmental discussion regarding diagnosis and surgical approach preceded the operation, a formal multidisciplinary consultation involving radiology and oncology was not undertaken. Besides, the limited understanding of primary mediastinal leiomyoma among clinicians is also a substantial contributing factor to the challenges in diagnosis. Therefore, clinicians should adopt systematic diagnostic approaches, such as checklists and differential diagnosis frameworks, to ensure comprehensive evaluation of potential conditions. And a multidisciplinary interaction between clinicians from different specialties appears crucial for an accurate diagnosis as well. Besides, continuous learning, expanding professional knowledge, and refining clinical experience are essential. By fostering awareness and evidence-based practices, clinicians can minimize the impact of cognitive fixedness, improving diagnostic precision and patient outcomes.

Primary mediastinal leiomyosarcoma is an aggressive malignant tumor characterized by a high risk of recurrence and metastasis, necessitating proactive treatment strategies. Surgical resection remains the cornerstone of therapy, particularly for localized disease, with the goal of achieving R0 resection. For patients for whom complete resection is unfeasible or who have metastatic lesions, adjuvant radiotherapy and chemotherapy are considered. The necessity of adjuvant therapy following R0 resection for primary mediastinal leiomyosarcoma remains controversial due to the rarity of the disease and limited evidence. A meta-analysis published in 2008, including soft-tissue sarcomas from all anatomical locations, demonstrated a slight survival benefit with postoperative doxorubicin and ifosfamide chemotherapy; however, it did not provide specific data on the subgroup of mediastinal leiomyosarcomas (9). On the contrary, Engelhardt et al. analyzed 976 patients diagnosed with mediastinal sarcoma, including 101 patients with primary mediastinal leiomyosarcoma, and concluded that there were no statistically significant differences in the adjusted overall survival regardless of whether adjuvant chemoradiotherapy was administered following radical resection (3). However, adjuvant therapy still be the potential treatment options in high-risk cases, such as those with large tumor size, high-grade histology, or close surgical margins. Consequently, to mitigate the risk of tumor progression in this patient, we elected to administer localized radiation therapy.

Targeted treatments and immunotherapy have experienced rapid development over the past two decades. However, unlike cancers driven by recurrent targetable mutations, such as BRAF V600E in melanoma, leiomyosarcomas typically exhibit a low tumor mutational burden and a paucity of actionable genetic alterations (10). The limited clinical utility of targeted therapies in leiomyosarcoma, attributable to its genomic profile, is evidenced by the unsatisfactory outcome of pazopanib—the only approved targeted agent for advanced soft tissue sarcoma—which yields an objective response rate of only 11% (11). Aside from pazopanib, targeted therapy options for leiomyosarcoma are limited to tissue-agnostic agents. The approved indications for these agents—such as pembrolizumab and dostarlimab—are restricted to tumors exhibiting high microsatellite instability or high tumor mutational burden (12, 13). However, immune checkpoint blockade has also shown limited efficacy in leiomyosarcomas, potentially due to the paucity of T-cell infiltration within the tumor microenvironment (14). In the future, it may be essential to employ a combination of therapies that targets multiple vulnerabilities in leiomyosarcomas, leveraging comprehensive and in-depth insights into the underlying molecular mechanisms of this disease.

## Conclusion

Primary mediastinal leiomyosarcomas are rare and aggressive soft tissue neoplasms that presents significant diagnostic and therapeutic challenges due to their rarity and high recurrence rate. In this study, through the analysis of a case of primary mediastinal leiomyosarcoma originating from the azygos vein, which was preoperatively misdiagnosed as a tracheal tumor, and supplemented by a literature review, we aim to enhance clinicians’ understanding of this rare disease, minimize diagnostic omissions in clinical practice, emphasize the importance of the multidisciplinary management.

## Data Availability

The raw data supporting the conclusions of this article will be made available by the authors, without undue reservation.
